# FGF signaling modulates mechanotransduction/WNT signaling in progenitors during tooth root development

**DOI:** 10.1038/s41413-024-00345-5

**Published:** 2024-06-24

**Authors:** Fei Pei, Tingwei Guo, Mingyi Zhang, Li Ma, Junjun Jing, Jifan Feng, Thach-Vu Ho, Quan Wen, Yang Chai

**Affiliations:** 1 Center for Craniofacial Molecular Biology, University of Southern California, 2250 Alcazar Street, CSA 103, Los Angeles, CA 90033 USA; 2 State Key Laboratory of Oral & Maxillofacial Reconstruction and Regeneration, Key Laboratory of Oral Biomedicine Ministry of Education, Hubei Key Laboratory of Stomatology, School & Hospital of Stomatology, Wuhan University, Wuhan, 430079 China

**Keywords:** Physiology, Bone

## Abstract

Stem/progenitor cells differentiate into different cell lineages during organ development and morphogenesis. Signaling pathway networks and mechanotransduction are important factors to guide the lineage commitment of stem/progenitor cells during craniofacial tissue morphogenesis. Here, we used tooth root development as a model to explore the roles of FGF signaling and mechanotransduction as well as their interaction in regulating the progenitor cell fate decision. We show that *Fgfr1* is expressed in the mesenchymal progenitor cells and their progeny during tooth root development. Loss of *Fgfr1* in *Gli1*^*+*^ progenitors leads to hyperproliferation and differentiation, which causes narrowed periodontal ligament (PDL) space with abnormal cementum/bone formation leading to ankylosis. We further show that aberrant activation of WNT signaling and mechanosensitive channel *Piezo2* occurs after loss of FGF signaling in *Gli1-Cre*^*ER*^*;Fgfr1*^*fl/fl*^ mice. Overexpression of *Piezo2* leads to increased osteoblastic differentiation and decreased *Piezo2* leads to downregulation of WNT signaling. Mechanistically, an FGF/PIEZO2/WNT signaling cascade plays a crucial role in modulating the fate of progenitors during root morphogenesis. Downregulation of WNT signaling rescues tooth ankylosis in *Fgfr1* mutant mice. Collectively, our findings uncover the mechanism by which FGF signaling regulates the fate decisions of stem/progenitor cells, and the interactions among signaling pathways and mechanotransduction during tooth root development, providing insights for future tooth root regeneration.

## Introduction

Stem/progenitor cells give rise to different cell lineages that contribute to organ development and morphogenesis.^1,2^ This process is guided by the physical microenvironment in which the stem/progenitor cells reside, including factors in the extracellular matrix, mechanical force, and more.^3,4^ These factors activate different signaling pathways, forming a signaling network to coordinate the migration, proliferation, and differentiation of stem/progenitor cells in tissue morphogenesis.

Tooth root development has been used as a model to explore the regulatory mechanism of the fate decisions of cranial neural crest (CNC)-derived progenitor cells. Progenitors in the dental papilla will form the dentin-pulp complex, and progenitors in the dental follicle are crucial for periodontal tissue.^5^ Abnormal cellular changes or behaviors of progenitor cells can lead to tooth root defects, such as shortened roots, dental dysplasia, and tooth ankylosis. Various signaling pathways and transcription factors are involved in regulating tooth root development and coordinating its morphogenesis, including BMP/TGFβ, WNT, SHH, and IGF signaling.^5–8^ FGF signaling is indispensable for craniofacial tissue development and morphogenesis. During embryonic tooth development (i.e., crown development), FGF signaling can be detected in the dental epithelium and CNC-derived mesenchyme, and plays a crucial role in tooth patterning and position determination.^9^ Lack of FGF signaling leads to arrested tooth germ development at the bud stage.^10^ Although the important roles of FGF signaling have been explored in different craniofacial organs, whether it participates in regulating tooth root development is still unknown. Furthermore, how FGF signaling achieves signaling specificity in regulating the organogenesis of different tissues also requires further investigation.

Stem/progenitor cells can sense their mechanical environment through cell-cell interactions, primary cilia, and mechanosensitive ion channels.^4,11^ During organogenesis, mechanical force modulates the fate of stem/progenitor cells; for example, it can activate TWIST expression and promote differentiation of the stomodeum and midgut tissue.^4,12^ Mechanical cues participate in many aspects of normal physiology and disease, so understanding the interplay between mechanical force and different signaling pathways in stem/progenitor cells is crucial for improving our understanding of organ morphogenesis, and could also shed light on the development of diseases.

In this study, we used the murine molar tooth roots as a model to study the role of FGF signaling and how it modulates mechanical force and other signaling to regulate progenitors’ fate decisions during organ morphogenesis. We discovered that *Fgfr1* is expressed in progenitor cells at the initiation of root development and gradually expands to the progeny of *Gli1*^+^ cells. Loss of *Fgfr1* in *Gli1*^+^ progenitor cells led to tooth ankylosis phenomena including a reduction in PDL space with hyperplastic cementum and abnormal bone-like tissue formation in *Gli1-Cre*^*ER*^*;Fgfr1*^*fl/fl*^ mice. These phenotypes were caused by hyperproliferation and differentiation of *Gli1*^+^ progenitor cells. We further showed that the mechanosensitive channel *Piezo2* is aberrantly activated to modulate WNT signaling to regulate the proliferation and differentiation of progenitors. By downregulating WNT signaling in *Fgfr1* mutant mice (*Gli1-Cre*^*ER*^*;Fgfr1*^*fl/fl*^;*β-catenin*^*fl/+*^), we were able to rescue the narrowed PDL space and abnormal cementum/bone formation seen in *Gli1-Cre*^*ER*^*;Fgfr1*^*fl/fl*^ mice. Our study illustrates how signaling pathways and mechanotransduction coordinate organ morphogenesis. Specifically, the FGF-PIEZO2-WNT signaling cascade regulates progenitor cell fate to control tooth root morphogenesis.

## Results

### *Fgfr1* is expressed in *Gli1*^+^ progenitor cells and their progeny during tooth root development

**Fig. 1:**
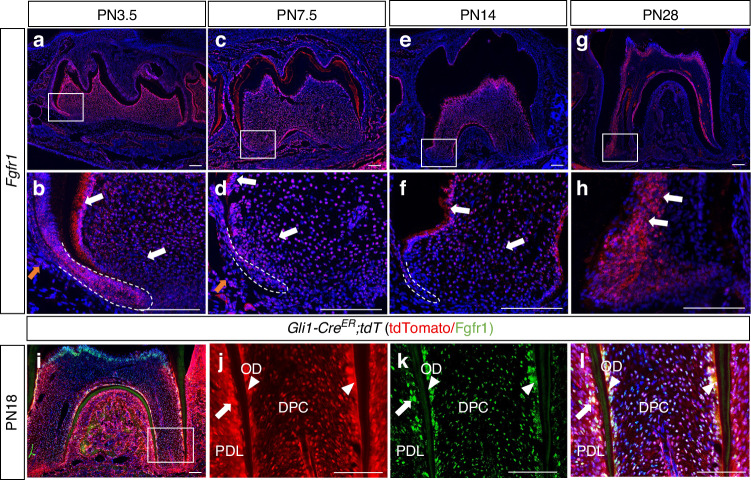
*Fgfr1* is expressed in *Gli1*^+^ progenitor cells and their progeny during tooth root development. **a**–**h** Expression of *Fgfr1* in mandibular first molars from wild-type mice at PN3.5, PN7.5, PN14 and PN28. White arrows point to the expression of *Fgfr1* in apical papilla and odontoblasts; yellow arrows point to the expression of *Fgfr1* in follicle cells. **i**–**l** Expression of *Fgfr1* and tdTomato at PN18 in *Gli1-Cre*^*ER*^*;tdT* mouse model. White arrows point to the expression of *Fgfr1* in periodontal ligament; white arrowheads point to the expression of *Fgfr1* in odontoblasts. PDL, periodontal ligament; OD, odontoblasts; DPC, dental pulp cells. White dashed lines outline Hertwig’s epithelial root sheath (HERS). Scale bars, 100 μm

Progenitor cells can give rise to different cell lineages and play important roles in organ development and morphogenesis. To investigate the role of *Fgfr1* in regulating *Gli1*^+^ progenitor cells, we analyzed its expression pattern during tooth root development. We found that it is widely expressed in the dental papilla at postnatal day (PN)3.5 and PN7.5, and can also be detected in the dental follicle and epithelial cells (Fig. 1a–d). Later, during tooth root development, the expression of *Fgfr1* was observed in odontoblasts, some dental pulp cells, and periodontium at PN14 and PN18 (Fig. 1e, f, i). At PN28, a more restricted pattern of *Fgfr1* was apparent in odontoblasts, which were differentiated and mature cells at this stage (Fig. 1g, h). We also found that, when we studied the progeny of *Gli1*^+^ cells labeled with tdTomato at PN18, colocalization between *Fgfr1* and tdTomato could be observed in the PDL, odontoblasts and a portion of the pulp cells (Fig. 1i–l). These results suggested that FGF signaling may play an important role in *Gli1*^+^ progenitor cells and their cell fate commitment during tooth root development.

### Loss of *Fgfr1* in *Gli1*^+^ progenitor cells leads to tooth ankylosis

**Fig. 2:**
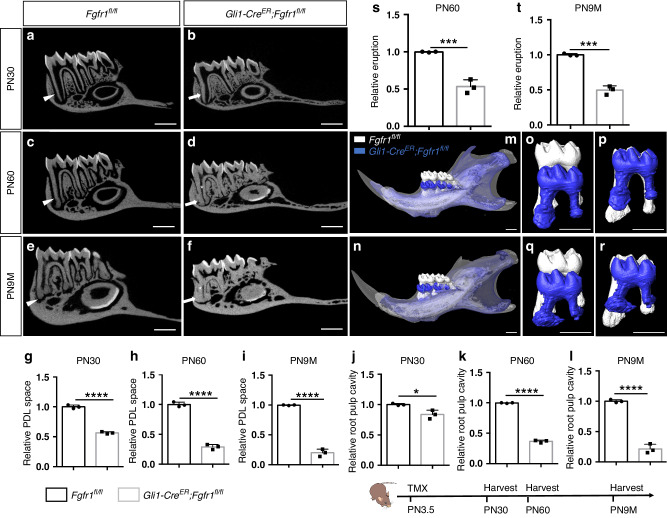
Loss of *Fgfr1* in *Gli1*^+^ progenitor cells leads to tooth ankylosis. **a**–**f** MicroCT analysis of the first mandibular molars in *Fgfr1*^*fl/fl*^ and *Gli1-Cre*^*ER*^*;Fgfr1*^*fl/fl*^ mice at PN30, PN60, and postnatal 9 months (PN9M). White arrowheads point to the periodontal ligament space; white arrows point to the narrowed periodontal ligament space; white asterisk points to the narrowed root pulp cavity. **g**–**i** Relative periodontal ligament space in control and *Fgfr1* mutant mice at PN30, PN60, and postnatal 9 months (PN9M). *P* < 0.000 1, unpaired Student’s t-test, *n* = 3 and each point represents one animal. **j**–**l** Relative tooth root pulp cavity area in control and *Fgfr1* mutant mice at PN30, PN60, and postnatal 9 months (PN9M). **j** *P* = 0.017 5, **k**, **l** *P* < 0.000 1, unpaired Student’s t-test, *n* = 3 and each point represents one animal. **m**–**n** MicroCT overlays: superimposition of control (white) and *Gli1-Cre*^*ER*^*;Fgfr1*^*fl/fl*^ (blue) mouse mandibles. **o**, **q** Overlay of the mandibular first molars of control (white) and *Gli1-Cre*^*ER*^*;Fgfr1*^*fl/fl*^ (blue) using the mandibular border as reference. **p**, **r** Overlay of the mandibular first molars of control (white) and *Gli1-Cre*^*ER*^*;Fgfr1*^*fl/fl*^ (blue) using the crown as reference. **s**, **t** Relative tooth eruption in control and *Fgfr1* mutant mice at PN60 and postnatal 9 months (PN9M). **s** *P* = 0.000 9, **t** *P* = 0.000 2. *n* = 3 and each point represents one animal, with an unpaired Student’s t-test performed. Schematic at the bottom indicates the induction protocol. *P < 0.05, ***P < 0.001, ****P < 0.000 1, Scale bars, 1 mm

To test the function of FGFR1 in regulating tooth root development, we deleted *Fgfr1* from the *Gli1*^+^ progenitors by generating *Gli1-Cre*^*ER*^*;Fgfr1*^*fl/fl*^ mice and injected them intraperitoneally at PN3.5 (the stage when tooth root development is initiated) with tamoxifen, through which we confirmed that *Fgfr1* expression was efficiently reduced (Fig. S1a–e). There was no obvious difference between control and *Fgfr1* mutant mice at PN14 based on histological analysis (Fig. S1f, g). We detected odontoblast and PDL differentiation with *Dspp* and periostin staining, respectively, which also showed no significant changes (Fig. S1h–k). After tooth root development, we found that the PDL space and dental pulp cavity were narrowed in *Gli1-Cre*^*ER*^*;Fgfr1*^*fl/fl*^ mice at PN30 (Fig. 2a, b, g, j), and this phenomenon was more severe by PN60 and postnatal 9 months (Fig. 2c–f, h, i, k, l). MicroCT analysis indicated that the apical root connected to the alveolar bone with narrowed PDL space and pulp cavity in *Fgfr1* mutant mice, which is similar to tooth ankylosis (Fig. 2d, f). Since tooth ankylosis leads to compromised physiological tooth movement, we further investigated the eruption of the first molar. We superimposed images of the mandibles of control and *Fgfr1* mutant mice according to a previous study,^13^ using the mandibular border as a reference structure. The results showed that tooth eruption was affected in *Gli1-Cre*^*ER*^*;Fgfr1*^*fl/fl*^ mice (Fig. 2m, n), while the size of the mandible was not significantly changed. The length of the tooth eruption relative to the mandibular border was compromised in *Gli1-Cre*^*ER*^*;Fgfr1*^*fl/fl*^ mice (Fig. 2m–t). The tooth root length was also found to be shorter in *Gli1-Cre*^*ER*^*;Fgfr1*^*fl/fl*^ mice when the teeth were superimposed at the crown region (Fig. 2p–r). These results suggested that the tooth roots were ankylosed to the mandible in *Gli1-Cre*^*ER*^*;Fgfr1*^*fl/fl*^ mice, which led to the impediment of eruption.

**Fig. 3:**
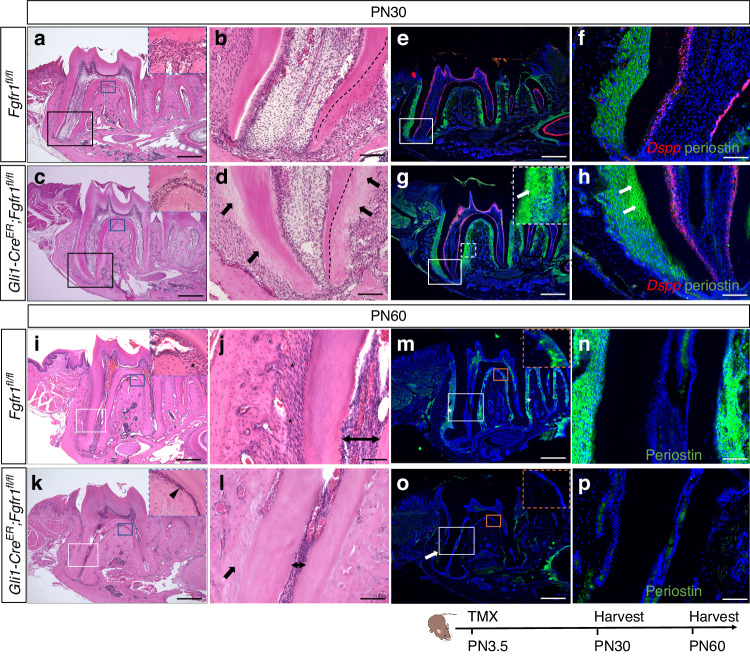
Narrowed PDL space with ankylosed tooth root in *Gli1-Cre*^*ER*^*;Fgfr1*^*fl/fl*^ mice. **a**–**d** Histological analysis of *Fgfr1*^*fl/fl*^ and *Gli1-Cre*^*ER*^*;Fgfr1*^*fl/fl*^ mice at PN30. Black arrows point to abnormal cementum. Black dashed lines outline the interface between root dentin and cementum. **e**–**h** Expression of *Dspp* and periostin in *Fgfr1*^*fl/fl*^ and *Gli1-Cre*^*ER*^*;Fgfr1*^*fl/fl*^ mice. White arrows point to abnormal periodontal ligaments. **i**–**l** Histological analysis of *Fgfr1*^*fl/fl*^ and *Gli1-Cre*^*ER*^*;Fgfr1*^*fl/fl*^ mice at PN60. The black asterisk points to periodontal ligament space; the black arrowhead points to narrowed periodontal ligament space in furcation; the black arrow points to the absence of periodontal ligament space where the tooth root connects to alveolar bone; Line with arrows indicates root pulp cavity. **m**–**p** Periostin expression in *Fgfr1*^*fl/fl*^ and *Gli1-Cre*^*ER*^*;Fgfr1*^*fl/fl*^ mice at PN60. The white asterisk points to the periodontal ligament; the white arrow points to the absence of periostin expression. The Schematic at the bottom indicates the induction protocol. Scale bars, **b**, **d**, **f**, **h**, **j**, **l**, **n**, **p**, 100 μm; others, 500 μm

Histological analysis showed cellular/acellular cementum and well-arranged PDL tissue in the molar roots of control mice at PN30 (Fig. 3a, b), while disordered mineralized tissue in the PDL was observed in the *Fgfr1* mutants (Fig. 3c, d). Interestingly, the main changes were detected in the acellular cementum. (Fig. 3c, d). By PN60, in the *Fgfr1* mutant mice the dental pulp cavity of the tooth root had narrowed significantly and the PDL in the apical root had almost disappeared, instead being filled with bone-like tissue connecting the root surface to the alveolar bone (Fig. 3k, l), which is a phenotype known as tooth ankylosis. These results suggested that the differentiation of progenitor cells may be affected in these mice. There was no obvious change in odontoblast differentiation revealed by *Dspp* staining at PN30 (Fig. 3e–h). To assess the character of the abnormal bone-like structure, we performed staining for the bone marker Sp7, which was detected in odontoblasts, cementoblasts, and alveolar bone in control mice, but not in the PDL (Fig. S2a, b). In the *Fgfr1* mutant mice, Sp7-labeled cementoblasts were detected along the surface of the tooth root, and Sp7 was strongly expressed in the disordered mineralized tissue in the PDL (Fig. S2c, d). This suggested that hyperplastic cementum and abnormal bone-like tissue were formed in *Gli1-Cre*^*ER*^*;Fgfr1*^*fl/fl*^ mice. By PN60, the PDL in these mice had barely detectable periostin staining (Fig. 3m–p). All these results demonstrated that *Fgfr1* in progenitor cells plays an important role in root-alveolar complex formation during tooth root development.

Since *Fgfr1* was also detected in the epithelium, we further tested whether loss of *Fgfr1* in epithelial progenitor cells may cause tooth ankylosis by generating *K14rtTA;teto-Cre;Fgfr1*^*fl/fl*^ mice. These mice showed normal cementum formation with well-arranged PDL tissue (Fig. S3a–d). Odontoblasts, PDL differentiation, and periodontal space were likewise unaffected in *K14rtTA;tetO-Cre;Fgfr1*^*fl/fl*^ mice (Fig. S3e–h). This suggested that the abnormal cementum and bone formation in *Gli1-Cre*^*ER*^*;Fgfr1*^*fl/fl*^ mice was not caused by the loss of *Fgfr1* in the dental epithelium. These results illustrated that *Fgfr1* in the dental mesenchymal progenitors plays an important role in regulating the root-alveolar complex formation during root development, the loss of which leads to abnormal cementum and bone formation with narrowed periodontal space.

### Loss of *Fgfr1* leads to hyperproliferation and differentiation of *Gli1*^+^ progenitor cells

**Fig. 4:**
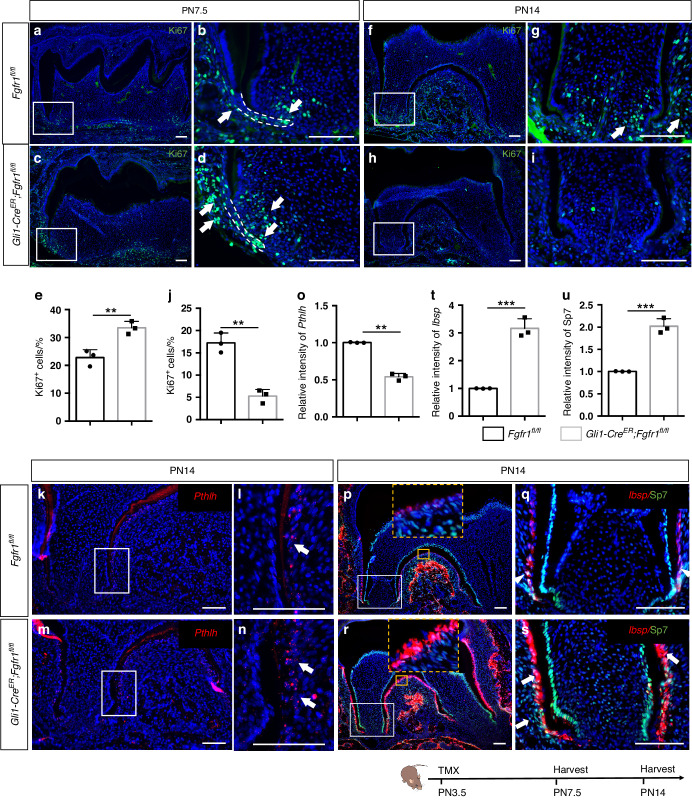
Loss of *Fgfr1* leads to hyperproliferation and differentiation of *Gli1*^+^ progenitor cells. **a**–**d**, **f**–**i** Proliferating cells stained with Ki67 in *Fgfr1*^*fl/fl*^ and *Gli1-Cre*^*ER*^*;Fgfr1*^*fl/fl*^ mice at PN7.5 and PN14. The white arrow points to Ki67^+^ cells in the papilla and follicle. **e**, **j** Quantification of Ki67^+^ cells in control and *Fgfr1* mutant mice at PN7.5 and PN14. **e** *P* = 0.007 3, **j** *P* = 0.001 5, *n* = 3, and each point represents one animal, with unpaired Student’s t-test performed. **k**–**n** Expression of *Pthlh* in control and *Fgfr1* mutant mice at PN14. The white arrow points to cementoblasts expressing *Pthlh* along the tooth root surface. **o** Quantification of *Pthlh*^+^ cells in control and *Fgfr1* mutant mice at PN14. *P* = 0.000 8, *n* = 3, and each point represents one animal, with unpaired Student’s t-test performed. **p**–**s** Expression of *Ibsp* and Sp7 in control and *Fgfr1* mutant mice at PN14. The white arrowhead points to cementoblasts expressing *Ibsp* and Sp7 along the tooth root surface; the white arrow points to increased *Ibsp* and Sp7 in cementoblasts and periodontium. **t**, **u** Quantification of *Ibsp*^+^ and Sp7^+^ cells in control and *Fgfr1* mutant mice at PN14. **t** *P* = 0.000 4, (**u**) *P* = 0.000 5, *n* = 3, and each point represents one animal, with unpaired Student’s t-test performed. The Schematic at the bottom indicates the induction protocol. **P < 0.01, ***P < 0.001, Scale bars, 100 μm

To investigate the underlying cellular mechanisms, we examined progenitor cell fate during tooth root development. Proliferation, assessed by Ki67 staining, was significantly increased in the apical and follicle cells surrounding Hertwig’s epithelial root sheath (HERS) in *Gli1-Cre*^*ER*^*;Fgfr1*^*fl/fl*^ mice at PN7.5 (Fig. 4a–e). Since the loss of *Fgfr1* in *Gli1*^+^ progenitor cells led to hyperproliferation, we generated *Gli1-Cre*^*ER*^*;Fgfr1*^*fl/fl*^*;Gli1-LacZ* mice to examine the number of *Gli1*^*+*^ progenitor cells. There was no obvious difference in the numbers of *Gli1*^*+*^ cells stained with β-Gal in control and *Fgfr1* mutant mice (Fig. S4a–e), which suggested that the self-renewal of *Gli1*^+^ progenitor cells was not affected. However, the proliferation decreased in *Fgfr1* mutant mice at PN14 (Fig. 4f–j). To determine the fate of these cells, we detected apoptosis with cleaved-caspase3 staining. There was no obvious change in apoptosis between control and *Fgfr1* mutant mice at either PN7.5 or PN14 (Fig. S5a–h), which suggested that these cells might instead be differentiating. We further detected cementoblasts with marker *Pthlh* and found its expression increased along the surface of the root in *Fgfr1* mutants (Fig. 4k–o). Upon analyzing the expression of osteoblast/cementoblast markers *Ibsp* and Sp7, we found that *Ibsp* was increased in cementoblasts on the root surface whereas Sp7 was increased in cementoblasts and cells in the PDL in *Fgfr1* mutants (Fig. 4p–u). These results indicated that loss of *Fgfr1* in *Gli1*^+^ progenitor cells causes the hyperproliferation of progenitors, which then differentiate and form abnormal cementum and bone-like structures in the PDL.

### Loss of FGF signaling in *Gli1*^+^ progenitor cells upregulates WNT signaling

**Fig. 5:**
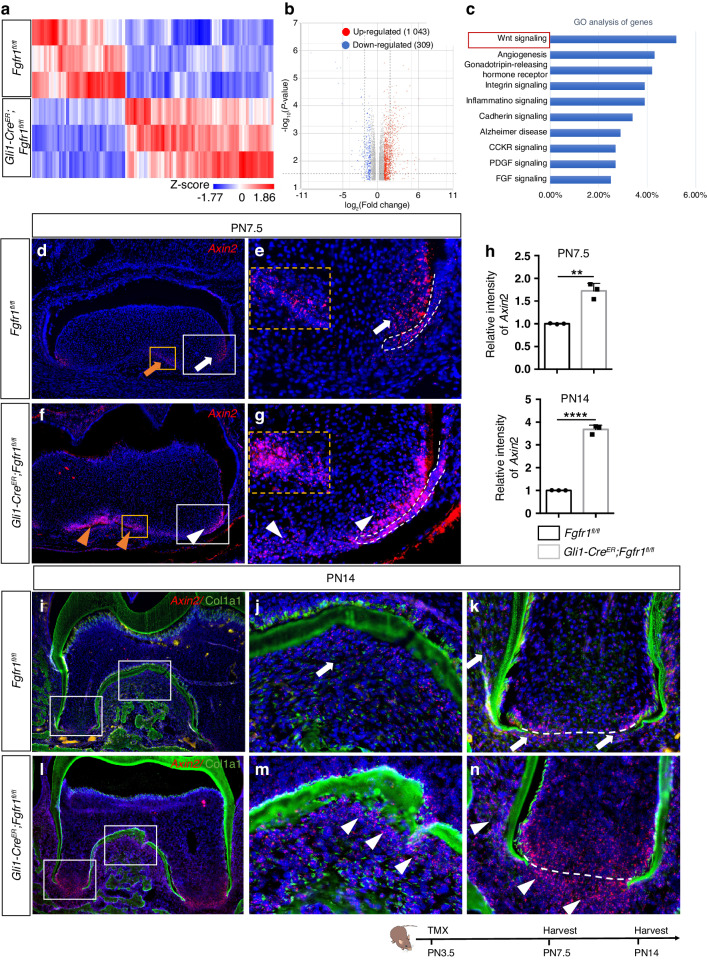
Loss of FGF signaling in tooth root mesenchymal progenitors leads to increased WNT signaling. **a** Hierarchical clustering showing the gene expression profiles of control and *Gli1-Cre*^*ER*^*;Fgfr1*^*fl/fl*^ mice. Z-scores were used to compare expression levels between samples. **b** Volcano plot showing 1 043 upregulated genes and 309 downregulated genes in *Fgfr1* mutant relative to control. **c** GO analysis showing the signaling pathways involved. **d**–**g** Expression of *Axin2* in *Fgfr1*^*fl/fl*^ and *Gli1-Cre*^*ER*^*;Fgfr1*^*fl/fl*^ mice at PN7.5. White and yellow arrows point to the expression of *Axin2* in the dental papilla surrounding HERS and furcation region; white and yellow arrowheads point to increased *Axin2* in the dental papilla, follicle, and furcation region. Orange boxes in d and f indicate furcation region and are shown enlarged in orange dashed insets in e and g. White boxes in (**d**) and (**f**) are shown enlarged as (**e**) and (**g**), respectively. White dashed lines outline HERS. **h** Relative fluorescent intensity of *Axin2* at PN7.5 and PN14. (PN7.5) *P* = 0.001 7, (PN14) *P* < 0.000 1, *n* = 3, and each point represents one animal, with unpaired Student’s t-test performed. **i**–**n** Expression of *Axin2* and Col1a1 in *Fgfr1*^*fl/fl*^ and *Gli1-Cre*^*ER*^*;Fgfr1*^*fl/fl*^ mice at PN14. White arrows point to the expression of *Axin2* in apical papilla and periodontium; white arrowheads point to the increased expression of *Axin2*. The white dashed line indicates the interface between the papilla and follicle. The Schematic at the bottom indicates the induction protocol. **P < 0.01, ****P < 0.000 1. Scale bars, 100 μm

To investigate how the FGF signaling regulates progenitor cell fate during development, we performed RNA-sequencing (RNA-seq) analysis using tissue from the apical region of control and *Gli1-Cre*^*ER*^*;Fgfr1*^*fl/fl*^ mouse first molars at PN7.5. Well-separated gene expression profiles distinguished the *Fgfr1* mutants from the controls (Fig. 5a). A total of 1 352 differentially expressed genes were found (>1.5-fold, *P* < 0.05), of which 1 043 were upregulated and 309 were downregulated in the *Fgfr1* mutants relative to the controls (Fig. 5b). Gene Ontology (GO) analysis showed that WNT signaling and FGF signaling were involved (Fig. 5c), which suggested that WNT signaling might be affected in *Gli1-Cre*^*ER*^*;Fgfr1*^*fl/fl*^ mice during tooth root development. We first investigated Axin2 and found that WNT signaling was increased in the apical papilla and follicle cells in *Gli1-Cre*^*ER*^*;Fgfr1*^*fl/fl*^ mice at PN7.5 (Fig. 5d–h). It was also upregulated in the apical papilla, periodontal tissue, and furcation region in *Fgfr1* mutant mice at PN14 (Fig. 5h–n). We further found that Frizzled Class Receptor 6 (FZD6), a receptor for WNT signaling proteins, was expressed in the apical papilla in control mice, but it was increased in apical and follicle cells in *Gli1-Cre*^*ER*^*;Fgfr1*^*fl/fl*^ mice at PN7.5 (Fig. S6a–d). We treated the mesenchymal cells from the apical region of the first molar of *Fgfr1* mutant mice with *Fzd6* siRNA (Fig. S6e, f), and found decreased *Ctnnb1* and impaired proliferation (Fig. S6g–j). This suggested that loss of *Fgfr1* in progenitor cells regulates proliferation via *Fzd6*/β-catenin, and that WNT signaling is upregulated after loss of FGF signaling in root progenitor cells.

### FGF signaling modulates mechanotransduction gene *Piezo2* to regulate WNT signaling and differentiation of progenitors

**Fig. 6:**
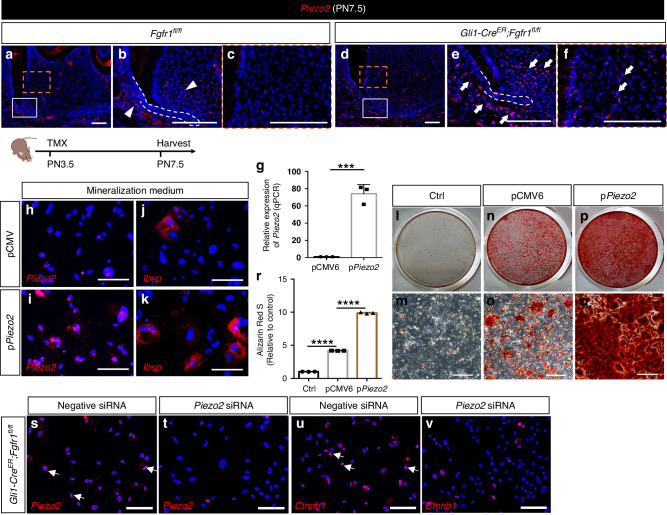
FGF signaling modulates mechanotransduction genes *Piezo2* to regulate differentiation of progenitors and WNT signaling. **a**–**f** Expression of *Piezo2* in *Fgfr1*^*fl/fl*^ and *Gli1-Cre*^*ER*^*;Fgfr1*^*fl/fl*^ mice at PN7.5. White arrowheads point to the expression of *Piezo2* in the apical papilla and follicle; white arrows point to increased *Piezo2* in the apical, middle, and coronal papilla and apical follicle. White dashed lines outline HERS. The Schematic at the bottom indicates the induction protocol. **g** Relative expression of *Piezo2* in pCMV6 and p*Piezo2*-treated group with qPRC in vitro. *P* = 0.000 3, unpaired Student’s t-test, *n* = 3 and each point represents one biological replicate. **h**–**k** Expression of *Piezo2* and *Ibsp* in pCMV6- and p*Piezo2*-treated apical mesenchymal cells from control mice. **l**–**q** Mineralized nodules with Alizarin red staining in control, pCMV6- and p*Piezo2*-treated apical mesenchymal cells from control mice. **r** Quantification of calcium deposition in the three groups. *P* < 0.000 1, one-way ANOVA, *n* = 3 biologically independent samples. **s**, **t** Knockdown of *Piezo2* in apical mesenchymal cells from *Gli1-Cre*^*ER*^*;Fgfr1*^*fl/fl*^ mice with *Piezo2* siRNA treatment. **u**, **v** Expression of *Ctnnb1* after *Piezo2* siRNA treatment. ***P < 0.001, ****P < 0.000 1. Scale bars, 100 μm

Mechanotransduction plays indispensable roles in bone homeostasis and remodeling,^14^ which led us to hypothesize that mechanotransduction could be involved in the abnormal cementum/bone-like tissue formation in *Gli1-Cre*^*ER*^*;Fgfr1*^*fl/fl*^ mice. We thus investigated the expression of *Piezo1* and *Piezo2*, two genes known to be involved in the relationship between signaling and mechanotransduction, in control and *Gli1-Cre*^*ER*^*;Fgfr1*^*fl/fl*^ mice. *Piezo1* showed no obvious change between controls and *Fgfr1* mutants in our RNA-seq results, whereas *Piezo2* was significantly upregulated in *Gli1-Cre*^*ER*^*;Fgfr1*^*fl/fl*^ mice (Fig. S7a, b). We verified their expression in vivo and found that *Peizo1* was widely expressed in the dental papilla and follicle cells in control mice with no obvious differences in the *Fgfr1* mutant mice (Fig. S7c–f). The expression of *Piezo2* was restricted to the apical papilla and follicle cells in control mice (Fig. 6a–c), with a similar expression pattern to that of the WNT signaling readout *Axin2*. However, *Piezo2* was increased in the apical papilla and follicle cells and showed ectopic expression in the middle and coronal papilla in *Gli1-Cre*^*ER*^*;Fgfr1*^*fl/fl*^ mice (Fig. 6d–f). This suggested that FGF signaling may regulate genes associated with mechanotransduction to modulate WNT signaling, thereby affecting cementum/bone formation during tooth root development. To verify the role of *Piezo2* in this signaling cascade, we overexpressed it in apical mesenchymal cells using *Piezo2* plasmid and confirmed efficient overexpression of *Piezo2* with qPCR analysis (Fig. 6g). Upon overexpression of *Piezo2* (Fig. 6h, i), we found that the osteogenic differentiation marker *Ibsp* was upregulated (Fig. 6j, k), and the number of mineralized nodules increased in *Piezo2* plasmid-transfected cells cultured in mineralization medium, as visualized by Alizarin Red S staining (Fig. 6l–r). We further downregulated *Piezo2* with siRNA in mesenchymal cells from the apical region of *Gli1-Cre*^*ER*^*;Fgfr1*^*fl/fl*^ mice to examine the WNT signaling pathway, and found the expression of *Ctnnb1* was decreased in *Fgfr1* mutant cells after *Piezo2* siRNA treatment (Fig. 6s–v). These results suggested that FGF signaling modulates *Piezo2* to regulate WNT signaling, thereby participating in the differentiation of *Gli1*^+^ progenitor cells. Taken together, our findings indicated that FGF signaling in *Gli1*^+^ progenitor cells modulates WNT signaling to regulate their proliferation and differentiation. Loss of FGF signaling in progenitor cells leads to increased expression of the mechanotransduction gene *Piezo2* and in turn, increases WNT signaling, which enhances abnormal bone formation during tooth root development.

**Fig. 7:**
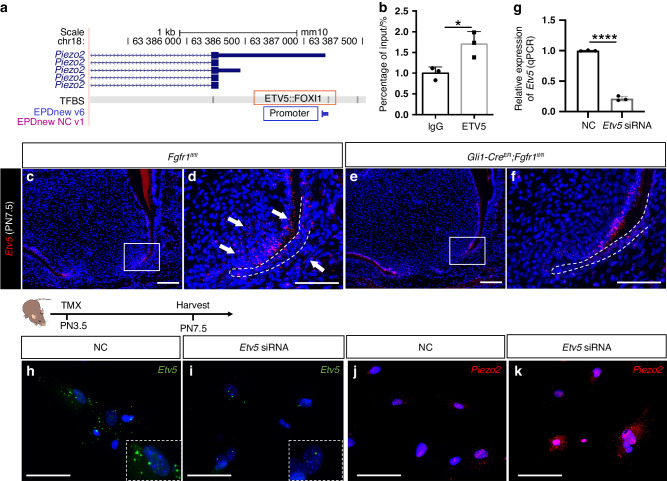
FGF/ETV5 signaling regulates *Piezo2* expression. **a** Prediction of ETV5 binding with *Piezo2* promoter region. **b** ChIP-qPCR showed that ETV5 can bind to the genomic locus of *Piezo2*. *P* = 0.023 1, unpaired Student’s t-test, *n* = 3, and each point represents one biological replicate. **c**–**f** Expression of *Etv5* in *Fgfr1*^*fl/fl*^ and *Gli1-Cre*^*ER*^*;Fgfr1*^*fl/fl*^ mice at PN7.5. White arrows point to the expression of *Etv5*. White dashed lines outline HERS. **g** Relative expression of *Etv5* in Negative control (NC) and *Etv5* siRNA-treated apical mesenchymal cells with qPRC in vitro. *P* < 0.000 1, unpaired Student’s t-test, *n* = 3 and each point represents one biological replicate. **h**, **i** Expression of *Etv5* in NC and *Etv5* siRNA-treated group. **j**, **k** Expression of *Piezo2* in NC and *Etv5* siRNA-treated group. *P < 0.05, ****P < 0.000 1. Scale bars, 100 μm

To investigate how FGF signaling regulates *Piezo2* expression, we analyzed the promoter region of *Piezo2* and predicted in silico that ETV5::FOXI1 could bind to this region (Fig. 7a). ChIP-qPCR using apical mesenchymal tissue confirmed that ETV5 can bind to the promoter region of *Piezo2* (Fig. 7b). *Etv5*, a downstream transcription factor of FGF signaling, was expressed in the apical mesenchyme adjacent to the dental epithelium and the follicle cells, and its expression decreased in *Gli1-Cre*^*ER*^*;Fgfr1*^*fl/fl*^ mice (Fig. 7c–f). This suggested that ETV5 might bind to the promoter region of *Piezo2* to suppress its expression. To test this, we downregulated *Etv5* with siRNA in mesenchymal cells from the apical region of control mice. The expression of *Piezo2* was upregulated after *Etv5* was efficiently decreased in apical mesenchymal cells with siRNA treatment (Fig. 7g–k). These results suggested that ETV5, as a downstream transcription factor of FGF signaling, binds to the promoter region of *Piezo2* to suppress its expression.

### Downregulation of WNT signaling rescues tooth ankylosis in *Gli1-Cre*^*ER*^*;Fgfr1*^*fl/fl*^ mice

**Fig. 8:**
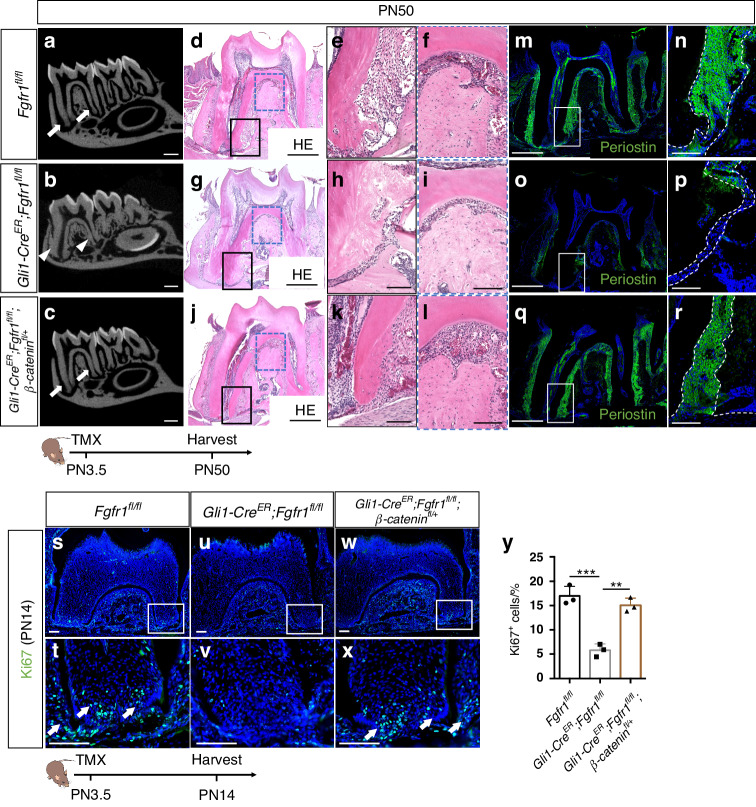
Downregulation of WNT signaling rescues tooth ankylosis in *Gli1-Cre*^*ER*^*;Fgfr1*^*fl/fl*^ mice. **a**–**c** MicroCT analysis of the first mandibular molars in *Fgfr1*^*fl/fl*^, *Gli1-Cre*^*ER*^*;Fgfr1*^*fl/fl*^ and *Gli1-Cre*^*ER*^*;Fgfr1*^*fl/fl*^;*β-catenin*^*fl/+*^ mice at PN50. White arrows point to the periodontal ligament space; white arrowheads point to the narrowed periodontal ligament space. **d**–**l** Histological analysis of *Fgfr1*^*fl/fl*^, *Gli1-Cre*^*ER*^*;Fgfr1*^*fl/fl*^ and *Gli1-Cre*^*ER*^*;Fgfr1*^*fl/fl*^;*β-catenin*^*fl/+*^ mice. **m**–**r** Periostin expression in *Fgfr1*^*fl/fl*^, *Gli1-Cre*^*ER*^*;Fgfr1*^*fl/fl*^ and *Gli1-Cre*^*ER*^*;Fgfr1*^*fl/fl*^;*β-catenin*^*fl/+*^ mice. Space between white dashed lines (**n**, **p**, and **r**) indicates periodontal ligament space. **s**–**x** Proliferation stained with Ki67 in *Fgfr1*^*fl/fl*^, *Gli1-Cre*^*ER*^*;Fgfr1*^*fl/fl*^ and *Gli1-Cre*^*ER*^*;Fgfr1*^*fl/fl*^;*β-catenin*^*fl/+*^ mice. White arrows point to Ki67^+^ cells. **y** Quantification of Ki67^+^ cells in three groups. *Fgfr1*^*fl/fl*^ versus *Gli1-Cre*^*ER*^*;Fgfr1*^*fl/fl*^: *P* = 0.000 4; *Gli1-Cre*^*ER*^*;Fgfr1*^*fl/fl*^ versus *Gli1-Cre*^*ER*^*;Fgfr1*^*fl/fl*^;*β-catenin*^*fl/+*^: *P* = 0.001, *n* = 3 biologically independent samples, with one-way ANOVA performed. The Schematic at the bottom indicates the induction protocol. **P < 0.01, ***P < 0.001. Scale bars, **a**–**c**, 1 mm; **d**, **g**, **j**, **m**, **o**, **q**, 500 μm; others, 100 μm

To test whether increased WNT signaling is responsible for abnormal cementum/bone formation in *Gli1-Cre*^*ER*^*;Fgfr1*^*fl/fl*^ mice, we downregulated WNT signaling in progenitor cells by generating a rescue model, *Gli1-Cre*^*ER*^*;Fgfr1*^*fl/fl*^;*β-catenin*^*fl/+*^ mice. To test the efficient decrease of WNT signaling, we examined Axin2 and *β-catenin* expression in our rescue mouse model and found that the levels of Axin2 and *β-catenin* were lower in the *Gli1-Cre*^*ER*^*;Fgfr1*^*fl/fl*^;*β-catenin*^*fl/+*^ mice than in *Gli1-Cre*^*ER*^*;Fgfr1*^*fl/fl*^ mice (Fig. S8a–g). Our analysis showed that the narrowed root PDL space seen in *Gli1-Cre*^*ER*^*;Fgfr1*^*fl/fl*^ mice were indeed rescued in *Gli1-Cre*^*ER*^*;Fgfr1*^*fl/fl*^;*β-catenin*^*fl/+*^ mice, as revealed by microCT analysis at PN50 (Fig. 8a–c). Histology showed that the abnormal cementum/bone in the apical root disappeared in the rescue model, and these mice exhibited normal cellular cementum with well-arranged PDL tissue (Fig. 8d, e, g, h, j, k). The narrowed PDL in the furcation region was also restored in *Gli1-Cre*^*ER*^*;Fgfr1*^*fl/fl*^;*β-catenin*^*fl/+*^ mice (Fig. 8f, i, l). Moreover, PDL differentiation defects were partially rescued in *Gli1-Cre*^*ER*^*;Fgfr1*^*fl/fl*^;*β-catenin*^*fl/+*^ mice (Fig. 8m–r). We further examined cellular changes after WNT signaling was downregulated in *Gli1-Cre*^*ER*^*;Fgfr1*^*fl/fl*^;*β-catenin*^*fl/+*^ mice, which showed that proliferation was restored in these mice at PN14 (Fig. 8s–y). These results suggested that the FGF-WNT signaling cascade plays an important role in tooth root development.

## Discussion

**Fig. 9:**
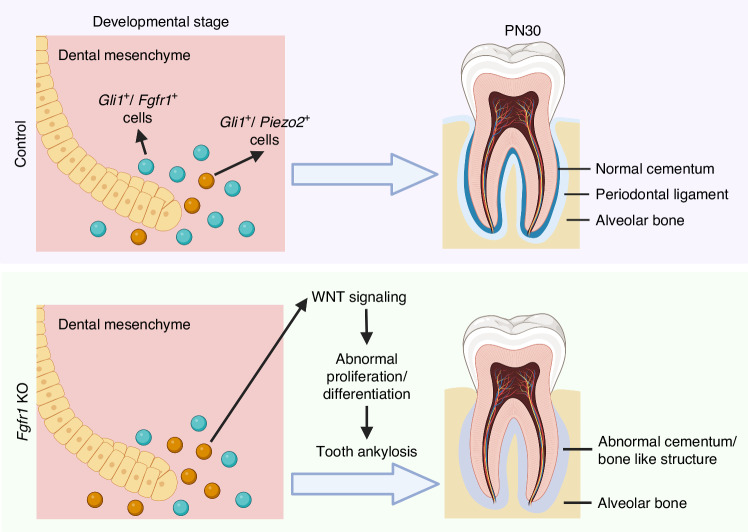
Schematic of FGF signaling in progenitor cells regulating mechanotransduction and WNT signaling to modulate proliferation and differentiation during tooth root development. Loss of Fgfr1 in *Gli1*^+^ progenitor cells leads to increased and ectopic *Piezo2* expression, which activates WNT signaling through Fzd6/*β-catenin* and *Piezo2*/*β-catenin*. Schematic was created with BioRender

Tooth root development is a dynamic process that involves cell-cell interactions to regulate the fate of progenitor cells.^5^ Multiple signaling molecules, transcription factors, and epigenetic regulators are known to play important roles in regulating tooth root development.^6,15,16^ Among the pathways involved, FGF signaling is particularly crucial. Moreover, mechanotransduction is an important factor in tissue development and morphogenesis.^4^ However, the roles of FGF signaling and mechanotransduction in tooth root morphogenesis are still unclear, as is the process by which they coordinate to achieve their functional specificity in regulating the fate of progenitors during organogenesis. Here, we investigated how FGF signaling regulates progenitor cell fate commitment and differentiation during postnatal tooth root development. In this study, we found that FGF signaling plays a crucial role in tooth root morphogenesis by modulating the proliferation and differentiation of *Gli1*^+^ progenitor cells. Loss of FGF signaling in *Gli1*^+^ progenitor cells led to tooth ankylosis in the *Gli1-Cre*^*ER*^*;Fgfr1*^*fl/fl*^ mouse model. Moreover, the mechanosensitive channel *Piezo2* was upregulated, which led to increased WNT signaling after the loss of FGF signaling (Fig. 9). We illustrated the crosstalk between signaling pathways and mechanotransduction during tooth root morphogenesis and further showed that an FGF/PIEZO2/WNT signaling cascade modulates progenitor cell fate to achieve FGF signaling specificity in regulating tooth root development.

FGF signaling has broad significance in the development and morphogenesis of multiple craniofacial tissues, including the craniofacial bones,^17,18^ palate^19,20^, and tooth crown.^9,21^ Although FGF signaling has been studied in tooth crown development, the landscapes of FGF ligand and receptor expression are different in embryonic and postnatal tooth development. For example, *Fgf10* is no longer present after the initiation of root development.^22,23^ This suggests that FGF signaling may have different roles and mechanisms during tooth root development. We found in the present study that FGF signaling is crucial for the formation of cementum and alveolar bone as well as for maintaining the fibrous PDL during tooth root development. Loss of FGF signaling in *Gli1*^*+*^ progenitors leads to tooth ankylosis. This suggests that FGF signaling is crucial for the osteogenic differentiation of stem/progenitor cells. Meanwhile, the mutation of FGF receptors can cause the fusion of cranial sutures associated with several types of craniosynostosis, such as Pfeiffer, Apert, and Crouzon syndromes.^18,24^ A recent study showed that nerve-derived FGF signaling is indispensable for stem cell maintenance in the mouse incisor.^25^ Loss of FGF ligand in nerves or FGFR1 in MSCs leads to abnormal dentin formation in the mouse incisor. These converging lines of evidence suggest that FGF signaling is crucial for the lineage commitment and fate decisions of stem and progenitor cells in the regulation of organogenesis in a variety of craniofacial tissues.

Signaling networks are crucial for organ morphogenesis. WNT, IGF and BMP signals have been found to regulate stem cells in spatially and temporally specific ways to modulate tooth root development. We have shown that loss of FGF signaling in progenitors upregulates WNT signaling and causes hyperproliferation and differentiation, which indicates the signaling pathways coordinate the tooth root morphogenesis. It is known that elevation of WNT/β-catenin signaling leads to abnormal mineralization of the PDL.^26^ Moreover, activation of β-catenin in osteoblasts and periodontium leads to increased bone formation and aberrant dento-alveolar complex formation.^27^ These results are consistent with our study’s findings. In our study, we found ectopic expression of *Fzd6* in the dental papilla, which affected cell proliferation. A previous study also showed that ectopic FZD6 expression can induce cell proliferation and sphere formation in proneural glioblastoma.^28^ *Fzd6* regulates both canonical and non-canonical WNT pathways,^28,29^ and here we found it can affect β-catenin during tooth root development, but whether this is a direct or indirect effect still remains to be explored in the future. While our work focused on what happens when loss of FGF signaling leads to upregulation of WNT signaling, previous studies have shown that disrupting WNT signaling also causes tooth root defects and disease. Disturbing WNT signaling in mesenchymal cells during tooth root development causes shortened roots with proliferation and differentiation defects.^30–33^ Taken together, these findings demonstrate that the level of WNT signaling must be tightly controlled for tooth root development to proceed normally. We further established the role of the FGF-WNT signaling cascade in establishing the correct tooth root pattern by modulating the fate commitment of progenitors.

During organ morphogenesis, cells receive and respond to mechanical forces from their external and internal environments.^34^ Mechanotransduction can play different roles depending on the type of cells involved and their context. In this study, we showed that the mechanosensitive channel *Piezo2* was downregulated by the FGF/ETV5 signaling pathway, while *Piezo1* was apparently unaffected. Previous study shows that both *Piezo1* and *Piezo2* are expressed in bone, and *Piezo1* shows a higher mRNA level than *Piezo2* in osteoblasts and osteocytes.^14^ We show that *Piezo1* is widely expressed in the dental papilla, while *Piezo2* is restricted to progenitor cells and involved in cementoblastic/osteogenic differentiation. This suggests that *Piezo1* and *Piezo2* may have different roles in tooth root development, which needs further study. *Piezo1/2* mediates mechanotransduction to regulate bone formation through β-catenin, and loss of *Piezo1* and *Piezo2* in osteoblast progenitor cells leads to decreased osteoblast differentiation.^35^ This is consistent with our study, and taken together, these findings suggest that the mechanical force/β-catenin signaling cascade is crucial for progenitor differentiation in different organs.

In summary, our study reveals that *Fgfr1* mutation can be responsible for tooth ankylosis, and further shows that FGF signaling regulates progenitor cell fate during tooth root morphogenesis via the FGF-PIEZO2-WNT signaling axis. This finding improves our understanding of the signaling pathway that governs CNC-derived progenitor cell lineage commitment during tooth root development and offers crucial information on how to control progenitor cells in tissue regeneration.

## Materials and methods

### Animals

*Gli1-Cre*^*ER*^ (JAX# 007913),^36^ tdTomato (JAK# 007905), *Fgfr1*^*fl/fl*^ (from Dr. Philippe Soriano),^37^ *K14rtTA* (JAX# 007678),^38^ *Teto-Cre* (JAX# 006234),^39^ and *β-catenin*^*fl/fl*^ (JAX# 004152)^40^ mouse lines were used in this study. The primers for genotyping were designed according to protocols from Jackson Labs using Integrated DNA Technologies products. All mice were housed in pathogen-free conditions. All animal studies were approved by the Institutional Animal Care and Use Committee (IACUC) at the University of Southern California (USC).

### Tamoxifen and doxycycline administration

Tamoxifen (Sigma, T5648) was dissolved in corn oil (Sigma, C8267) at 20 mg/mL. *Fgfr1*^*fl/fl*^, *Gli1-Cre*^*ER*^*;Fgfr1*^*fl/fl*^ and *Gli1-Cre*^*ER*^*;Fgfr1*^*fl/fl*^;*β-catenin*^*fl/+*^ mice were injected once intraperitoneally at a dosage of 1.5 mg/10 g body weight at PN3.5. Doxycycline rodent diet was fed to dams of *K14rtTA;tetO-Cre;Fgfr1*^*fl/fl*^ mice (Envigo, TD.08541) every day beginning when the suckling pups were at PN3.5. A dosage of 50 mg/mL doxycycline (Sigma-Aldrich; D9891) was injected into the pups intraperitoneally at PN3.5 and PN5.5.

### MicroCT analysis

We collected mandibles from mice 30 days, 50 days, 60 days, and 9 months of age and then fixed them with 4% paraformaldehyde, and mandibles from more than three mice were collected for each group. MicroCT analysis was performed using a Skyscan 1174v1.2 (Bruker Corporation, USA) at 50 kVp, 800 μA and a resolution of 16.7 mm, followed by visualization and three-dimensional reconstruction performed using Avizo/Amira 9.5.0 (Visualization Sciences Group, France).

### Histological analysis

Mouse mandibles were fixed in 4% paraformaldehyde (PFA) overnight after dissection. After 10% EDTA treatment for 2–4 weeks, the samples were decalcified and then dehydrated in an ethanol and xylene series. The samples were embedded in paraffin and cut into 6-7 μm sections using a microtome (Leica). H&E staining was performed according to standard protocols. We performed serial sectioning to make sure different levels were captured and checked every section of both control, *Fgfr1* mutant, and *Gli1-Cre*^*ER*^*;Fgfr1*^*fl/fl*^;*β-catenin*^*fl/+*^ rescue samples. All staining protocols in this study used this method.

### In situ hybridization

For cryosections, sections were stained according to the manufacturer’s instructions using an RNAscope Multiplex Fluorescent v2 kit (Advanced Cell Diagnostics, 323100). For cells, staining was performed after fixation with 10% Neutral Buffered Formalin (NBF) for 30 min. All probes used in this study were synthesized by Advanced Cell Diagnostics: Probe-Mm-*Fgfr1* (454941), Probe-Mm-*Dspp* (448301), Probe-Mm-*Fzd6* (404921), Probe-Mm-*Piezo2* (400191), Probe-Mm-*Piezo1* (500511), Probe-Mm-*Axin2* (400331), Probe-Mm-*Ibsp* (415501), Probe-Mm-*Pthlh* (456521), and Probe-Mm-*Ctnnb1* (311741).

### Immunofluorescence

The decalcified samples were dehydrated in serial sucrose solutions and then embedded in an optimal cutting temperature compound (Tissue-Tek). The samples were cut into 8 μm cryosections using a cryostat (Leica CM1850). The cryosections were treated with a blocking solution (PerkinElmer) for 1 h. The primary antibodies used were the following: Sp7 (Abcam; ab209484, 1:100), Periostin (1:100, Abcam, ab14041), Ki67 (1:100, Abcam, ab15580), Col1a1 (1:100, CST, 72026), and Cleaved Caspase3 (9661, Cell signaling, 1:200 with TSA). After being incubated with primary antibodies at 4 °C overnight, signals were detected with Alexa-conjugated secondary antibody (1:200, Invitrogen), and nuclei were stained with DAPI (Invitrogen, 62248). Images were captured with a Keyence microscope (Carl Zeiss).

### RNA sequencing

First mandibular molars from the control and *Gli1-Cre*^*ER*^*;Fgfr1*^*fl/fl*^ mice were dissected at PN7.5 after tamoxifen induction. The apical region of the molar was collected following RNA extraction with an RNeasy Micro Kit (Qiagen, 74004). For RNA-sequencing analysis, cDNA library preparation and sequencing were performed on NextSeq500 High Output equipment for three pairs at the Technology Center for Genomics & Bioinformatics at the University of California, Los Angeles (UCLA). Raw reads were trimmed, aligned with the mm10 genome, and normalized in Partek Flow. Differential analysis was performed by selecting transcripts with a significance of *P* < 0.05.

### Plasmid transfection and qPCR

Plasmids were from OriGene, including pCMV6-AC-GMP (PS100010) and Piezo2 (NM_001039485 Mouse Tagged ORF Clone, MR226955). Plasmid transfection was performed following the manufacturers’ protocols (QIAGEN, 301704, and OriGene, TF81001). Briefly, with 1 µg/µL stock solution, the plasmid was transfected into cells in 24-well plates for 2 days followed by real-time qPCR.

The total RNA was isolated using RNeasy Plus Micro Kit (QIAGEN, 74034) after cells were collected. cDNA transcription was performed using iScript™ cDNA Synthesis Kit (Bio-495 Rad, 1708891). qPCR quantification was performed using SsoFast™ EvaGreen® Supermix (Bio-Rad, 1725202) on a Bio-Rad CFX96 Real-Time System. The primer sequences used in this study were as follows: *Gapdh* (forward primer 5′-AGGTCGGTGTGAACGGATTTG-3′, reverse primer 5′-TGTAGACCATGTAGTTGAGGTCA-3′), *Piezo2* (forward primer 5′- TCAACTGCTCCTTGCCCAAT-3′, reverse primer 5′-ATGGCGGTAAACGGTGACTT-3′), *Etv5* (forward primer 5′- TCAGTCTGATAACTTGGTGCTTC-3′, reverse primer 5′-GGCTTCCTATCGTAGGCACAA-3′).

### Cell culture and osteogenic differentiation

The apical mesenchymal tissues of the first mandibular molars from control and *Fgfr1* mutant mice were collected at PN3.5, then cut into small pieces and cultured in α-MEM (Thermo Fisher, 12571071) with 10% FBS (Thermo Fisher, 12662029) at 37 °C in a 5% CO_2_ incubator.

StemPro osteogenesis differentiation kit (Thermo Fisher, A1007201) was used for the osteogenic differentiation following the manufacturer’s protocol.

### Alizarin Red S staining and quantification

Cells were washed twice with PBS after removing the medium, then fixed with 4% paraformaldehyde for 30–40 min. After washing with PBS twice, cells were stained with 0.2% Alizarin Red S solution for 15 min and then washed with PBS to remove unspecific staining. Calcified nodules stained with red were photographed with a microscope. To quantify the calcium deposition, the stain was solubilized with 10% cetylpyridinium chloride monohydrate (CPC, Sigma-Aldrich) in 0.1 mol/L PBS (pH 7.0) for 15 min. The absorbance was read at 570 nm.

### siRNA transfection

Cells were passaged for siRNA transfection when they reached sub-confluence. AllStars Negative Control siRNA (QIAGEN,1027280), Etv5 siRNA (QIAGEN, 1027416, Mm_Etv5_1 FlexiTube siRNA SI00996744; Mm_Etv5_2 FlexiTube siRNA SI00996737; Mm_Etv5_3 FlexiTube siRNA SI00996730; Mm_Etv5_4 FlexiTube siRNA SI00996723), Piezo2 siRNA (QIAGEN, 1027416, Mm_Piezo2_1 FlexiTube siRNA SI04723404; Mm_Piezo2_2 FlexiTube siRNA SI04723397; Mm_Piezo2_3 FlexiTube siRNA SI04723390; Mm_Piezo2_4 FlexiTube siRNA SI04723383), Fzd6 siRNA (QIAGEN, 1027416, Mm_Fzd6_1 FlexiTube siRNA SI02708510; Mm_Fzd6_2 FlexiTube siRNA SI02686684; Mm_Fzd6_3 FlexiTube siRNA SI02666979; Mm_Fzd6_4 FlexiTube siRNA SI00171451), Opti-MEM I Reduced Serum Medium (Thermo Fisher, 31985062) and lipofectamine™ RNAiMAX (Thermo Fisher, 13778075) were used in this study. SiRNA was transfected into cells at a final concentration of 10 nmol/L.

### ChIP-qPCR

The mandibular first molars were dissected from wild-type mice at PN3.5. 60–80 mg tissue from multiple animals was combined for each replicate. Samples were prepared for ChIP following the manufacturer’s protocol (Chromatrap, 500191). Briefly, tissue was cut into small pieces, fixed with 1% formaldehyde at room temperature for 15 min, and incubated with 0.65 mol/L glycine solution. After washing with PBS twice, the tissue was resuspended in Hypotonic Buffer and incubated at 4°C for 10 min to obtain nuclei. The pellet was resuspended using Digestion Buffer. Chromatin was sheared into 100–500 bp fragments with Shearing Cocktail. 10 µg chromatin with ETV5 antibody (Proteintech, 13011-1-AP) or immunoglobulin G-negative control was added to the Column Conditioning Buffer. Immunoprecipitation (IP) slurry was mixed thoroughly and incubated on a rotor for 1 h at 4 °C. An equivalent amount of chromatin was set as an input. A chromatrap spin column was used to purify the IP slurry at room temperature, and chromatin was eluted using the ChIP-seq elution buffer. The chromatin sample and input were further incubated at 65°C overnight to reverse cross-linking. DNA was treated with proteinase, then purified with the Chromatrap DNA purification column. Primers were designed using the promoter region of *Piezo2*. Input, negative control, and ChIP eluates were assayed using real-time qPCR. Primers were designed using the promoter region of *Piezo2*. Forward: 5’- CGCTCCCAGGAAATGTTCTCTG-3’; Reverse: 5’-GCTATGTCTCCACGTAGGCATCT-3’.

### Western blot

First mandibular molars from *Fgfr1*^*fl/fl*^ control, *Gli1-Cre*^*ER*^*;Fgfr1*^*fl/fl*^ mutant and *Gli1-Cre*^*ER*^*;Fgfr1*^*fl/fl*^;*β-catenin*^*fl/+*^ rescue mice were dissected at PN8.5 after tamoxifen induction. The apical region of the molar was collected and incubated in RIPA buffer (Cell Signaling, 9806) for 30 min, then centrifuged at 14 000 × *g* at 4 °C. Total protein was loaded in 4%–15% precast polyacrylamide gel and transferred to PVDF membranes. After blocking for 1 h, samples were incubated with primary antibody against beta Catenin (1:100, Abcam, ab6302) at 4 °C overnight, and detected with secondary antibodies on an Azure 300 (Azure Biosystems).

### Statistical analysis

Statistical analysis was performed with GraphPad Prism. All statistical data are presented as individual points and mean ± SD. Unpaired Student’s t-test or one-way ANOVA analysis was used for comparisons, with *P* < 0.05 considered statistically significant. *n* ≥ 3 for all experiments.

### Supplementary information

Supplemental material

Source file

## Data Availability

Bulk RNA-seq datasets are available through the GEO database under accession code GSE233576 (token: azqvquymndkvnwh).
