# Supplementary material for: FGF signaling modulates mechanotransduction/WNT signaling in progenitors during tooth root development
Source: Bone Res. 2024 Jun 24;12:37. doi: 10.1038/s41413-024-00345-5 (PMC11194271; doi:10.1038/s41413-024-00345-5)
Supplement: Supplementary file 1 — Supplemental material [file 41413_2024_345_MOESM1_ESM.docx]

**FGF signaling modulates mechanotransduction/WNT signaling in progenitors during tooth root development**

This document includes:

**Supplementary Fig.1 to Supplementary Fig. 8**

Supplementary Fig. 1

**Supplementary Fig. 1:** No obvious differences were detected between control and *Gli1-Cre^ER^;Fgfr1^fl/fl^* mice at PN14. **a-d** Expression of *Fgfr1* in *Fgfr1^fl/fl^* and *Gli1-CreER;Fgfr1^fl/fl^* mice at PN7.5. **e** Quantification of *Fgfr1* in control and mutant mice at PN7.5. P<0.0001, n=3 and each point represents one animal, with unpaired Student’s t-test performed. **** P<0.0001.  **f-g** Histological analysis of *Fgfr1^fl/fl^* and *Gli1-Cre^ER^;Fgfr1^fl/fl^* mice at PN14. **h-i** *Dspp* expression in *Fgfr1^fl/fl^* and *Gli1-Cre^ER^;Fgfr1^fl/fl^* mice. **j-k** Periostin expression in *Fgfr1^fl/fl^* and *Gli1-Cre^ER^;Fgfr1^fl/fl^* mice at PN14.

Supplementary Fig. 2

**Supplementary Fig. 2:** Loss of *Fgfr1* in *Gli1*^+^ progenitor cells led to abnormal bone-like tissue formation at PN30. **a-d** Expression of Sp7 in *Fgfr1^fl/fl^* and *Gli1-Cre^ER^;Fgfr1^fl/fl^* mice. White asterisk points to periodontal ligament; white arrows point to abnormal expression of Sp7 in periodontal ligament space along root surface. Scale bars, **b**, **d**, 100 μm; others, 500 μm.

Supplementary Fig. 3

**Supplementary Fig. 3:** Loss of *Fgfr1* in the epithelium did not lead to tooth ankylosis. **a-d** Histological analysis of *Fgfr1^fl/fl^* and *K14rtTA;tetO-Cre;Fgfr1^fl/fl^* mice. **e-h** Expression of *Dspp* and periostin in *Fgfr1^fl/fl^* and *K14rtTA;tetO-Cre;Fgfr1^fl/fl^* mice. Schematic at the bottom indicates induction protocol. Scale bars, **b**, **d**, **f**, **h**, 100 μm; others, 500 μm.

Supplementary Fig. 4

Supplementary Fig. 4: **a-d** Expression of Gli1+ cells in *Fgfr1^fl/fl^* and *Gli1-CreER;Fgfr1^fl/fl^* mice. Boxes in **a-b** are magnified in **c-d** respectively. Dotted lines in c-d indicate dental epithelium. **e** Quantification of *Gli1^+^* cells in *Fgfr1^fl/fl^* and *Gli1-CreER;Fgfr1^fl/fl^* mice. ns, not significant; n=3 and each point represents one animal, with unpaired Student’s t-test performed. Scale bars: 100 µm.

Supplementary Fig. 5

**Supplementary Fig. 5:** No significant changes in apoptosis were detected between *Fgfr1^fl/fl^* and *Gli1-CreER;Fgfr1^fl/fl^* mice. **a-h** Apoptosis detected with cleaved-caspase3 staining in *Fgfr1^fl/fl^* and *Gli1-CreER;Fgfr1^fl/fl^* mice at PN7.5 and PN14. Schematic at the bottom indicates induction protocol. Scale bars, 100 μm.

Supplementary Fig. 6

**Supplementary Fig. 6:** Loss of *Fgfr1* in *Gli1*^+^ progenitor cells led to increased expression of *Fzd6* in follicle as well as apical, middle and coronal papilla. **a-d** Expression of *Fzd6* in *Fgfr1^fl/fl^* and *Gli1-Cre^ER^;Fgfr1^fl/fl^* mice at PN7.5. White arrows point to increased expression of *Fzd6* in dental follicle and papilla. White dashed lines outline HERS. Schematic at the bottom indicates induction protocol. **e-j** Knockdown of *Fzd6* in cells from mutant mice with siRNA treatment led to decreased Ki67 and *Ctnnb1*. **e-f** *Fzd6* was knocked down with siRNA treatment. **g-j** Expression of *Ctnnb1* and Ki67. Arrows indicate representative positive signals. Scale bars, 100 μm.

Supplementary Fig. 7

**Supplementary Fig. 7:** No obvious difference in *Piezo1* was detected in *Fgfr1^fl/fl^* and *Gli1-CreER;Fgfr1^fl/fl^* mice at PN7.5. **a-b** *Piezo2* was increased in *Gli1-CreER;Fgfr1^fl/fl^* mice, while *Piezo1* exhibited no significant change in RNAseq analysis. **c-****f** Expression of *Piezo1* in *Fgfr1^fl/fl^* and *Gli1-Cre^ER^;Fgfr1^fl/fl^* mice at PN7.5. Schematic at the bottom indicates induction protocol. Scale bars, 100 μm.

Supplementary Fig. 8

**Supplementary Fig. 8: a-f** Expression of *Axin2* in control mice, *Fgfr1* mutant mice, and rescue model. Boxes in **a-c** are magnified in **d-f** respectively. Asterisks in (**d**) and (**f**) indicate decreased expression of *Axin2* compared to the *Fgfr1* mutant mice. Dotted lines in D-F indicate dental epithelium. Scale bars: 100 µm. **g** Western blot of β-catenin in the three groups.
